# Transcriptomic Analysis Elaborates the Resistance Mechanism of Grapevine Rootstocks against Salt Stress

**DOI:** 10.3390/plants11091167

**Published:** 2022-04-26

**Authors:** Fanggui Zhao, Ting Zheng, Zhongjie Liu, Weihong Fu, Jinggui Fang

**Affiliations:** 1 College of Horticulture, Nanjing Agricultural University, Nanjing 210095, China; zhaofgui@163.com (F.Z.); zhengting@njau.edu.cn (T.Z.); 2017204011@njau.edu.cn (Z.L.); 2020204012@stu.njau.edu.cn (W.F.); 2 Institute of Horticulture, Zhejiang Academy of Agricultural Sciences, Hangzhou 310021, China

**Keywords:** grape, rootstock, salt stress, signal transduction

## Abstract

Grapes are subject to a wide range of climatic conditions during their life cycle, but the use of rootstocks can effectively ameliorate the effects of abiotic stress. However, the tolerance mechanism of different grape rootstock varieties varies under various stresses, and systematic research on this aspect is limited. On the basis of previous research, transcriptome sequencing was performed on three tolerant grape rootstock varieties (3309C, 520A, 1103P) and three intolerant grape rootstock varieties (5BB, 101–14, Beta). In total, 56,478,468 clean reads were obtained. One hundred and ten genes only existed in all combinations during P1 with a downregulated trend, and 178 genes existed only in P1 of tolerant grape rootstock varieties. Salt treatment firstly affected the photosynthesis of leaves, and tolerant varieties weakened or even eliminated this effect through their own mechanisms in the later stage. Tolerant varieties mobilized a large number of MFs during the P2 stage, such as hydrolase activity, carboxypeptidase activity, and dioxygenase activity. Carbon metabolism was significantly enriched in P1, while circadian rhythm and flavonoid biosynthesis were only enriched in tolerant varieties. In the intolerant varieties, photosynthesis-related pathways were always the most significantly enriched. There were large differences in the gene expression of the main signal pathways related to salt stress in different varieties. Salt stress affected the expression of genes related to plant abiotic stress, biotic stress, transcription factors, hormones, and secondary metabolism. Tolerant varieties mobilized more bHLH, WRKY, and MYB transcription factors to respond to salt stress than intolerant varieties. In the tolerant rootstocks, SOS was co-expressed. Among these, SOS1 and SOS2 were upregulated, and the SOS3 and SOS5 components were downregulated. The genes of heat shock proteins and the phenylalanine pathway were upregulated in the tolerant varieties. These findings outline a tolerance mechanism model for rootstocks for coping with osmotic stress, providing important information for improving the resistance of grapes under global climate change.

## 1. Introduction

Grape (*Vitis vinifera* L.) is an economically important fruit crop that is mainly grown on a commercial scale in vineyards throughout the world. Grapes are mostly used for wine making and fresh food, or are processed into raisins, juice, color, tannins, and antioxidant products. During the cultivation process, drought (caused by either limited rainfall or insufficient groundwater supply), flooding (caused by either heavy rain or flooding), and soil salinization are significant stresses experienced by grapevines, posing a serious threat to grape productivity [1,2,3]. Prolonged exposure to salt stress conditions can cause serious damage to plants, including damage to the cell membranes, metabolic toxicity, the formation of large amounts of reactive oxygen species, reduced photosynthesis, and reduced nutrient absorption [4,5].

Salt stress in higher plants is regulated by a number of physiological and biochemical processes [6]. Plants have evolved complex regulatory networks and molecular mechanisms to respond appropriately to stress conditions in their living environment [7]. This regulatory network involves upstream signaling molecules, including hormones such as abscisic acid (ABA), reactive oxygen species (ROS), nitric oxide (NO), and calcium channels (Ca^2+^), as well as downstream transcriptional regulation by transcription factors [8]. Common molecular mechanisms include membrane lipid desaturation to reduce membrane damage, the induction of molecular chaperones to inhibit protein denaturation, the accumulation of osmotic substances to maintain osmotic pressure, and active oxygen scavenging and other conservative cellular defense methods. A large number of studies have found that many transcription factors, miRNAs and salt-sensitive genes can sense salt signals and regulate their expression levels to enhance or reduce a plant’s tolerance to salt, in which salt-sensitive genes are a class of genes that plants use to respond to external salt stress [4,9,10]. Salt Overly Sensitive 3 (SOS3) and sucrose non-fermenting1 (SNF1) related protein kinase 3 (SnRK3) were found to respond to the initial osmotic stress signal induced by salt stress [5]. The Na^+^/H^+^ antiporter NHX and VACUOLAR H^+^/-PYROPHOSPHATASE AVP can enhance the absorption capacity of root tissue cell vacuoles for Na^+^, thereby improving the ability of plants to adjust to osmotic stress [10]. The SOS pathway via the SOS2–SOS3 complex phosphorylates the H^+^/cation antiporter SOS1/NHX7, which can transport sodium out of the cell and plays an important role in salt stress adaptability [11,12]. In addition, many genes, such as ENHANCED RESPONSE TO ABA 1 (ERA1), protein phosphatase 2C (PP2C), and ABA activated protein kinase (AAPK), can reduce the accumulation of toxic ions in tissues by delaying stomatal closure when plants are under osmotic stress [13]. In addition to salt-sensitive genes that can directly respond to salt stress, transcription factors regulating the expression of these genes also play important roles in the process of plants responding to salt stress, such as AP2, MYB, bZIP, WRKY, NAC, etc. [9,10,14]. At present, research into the molecular mechanisms of plant salt tolerance has mainly focused on model crops such as *Arabidopsis thaliana*, while there are relatively few studies on perennial woody fruit tree crops. In grapes, there are many studies on the changes in the morphological characteristics and physiological and biochemical indicators, but there are only few reports on the dynamic changes in gene or protein expression profiles and functional studies of salt tolerance candidate genes [15,16,17,18]. Tak et al. [19,20] isolated *SDR1* and *bZIP23*, which are homologous to *Arabidopsis thaliana* and are involved in stress tolerance in grapes, and the functional verification results showed that both *VvSDR1* overexpressed in tobacco and *VvbZIP23* overexpressed in grape callus enhanced the resistance to salt stress.

The grafting of scion grapes onto suitable resistant rootstocks can significantly improve the interaction between grapes and the environment, and can reduce the impact of abiotic stress [21,22]. This may currently be the most effective way to deal with abiotic stress and reduce production losses [23,24,25]. Hence, interest in grape rootstocks has intensified. There is a wide variation for salt tolerance amongst rootstock genotypes [26,27]. Grape rootstocks vary in their response to soil salinity in terms of maintaining scion growth, sustaining yield, grape quality, and ion concentrations in the leaves [28,29,30]. Salinity tolerance may also involve exclusion of chloride and sodium ions [30,31]. Elucidating the stress resistance process of the rootstocks at the physiological and molecular levels will play an important role in improving crop growth performance under stress conditions [6]. Therefore, we selected the 10 most common grape rootstocks to analyze and evaluate the morphological physiological characteristics and antioxidant enzyme activities of plants under salt stress, and finally screened the tolerant varieties 3309C, 520A, and 1103P, and the intolerant varieties 5BB, 101-14, and Beta (Figure S1), which is consistent with viticulture and previous research findings [6,26,32,33]. In this study, we selected varieties with large differences in stress tolerance for analyzing the global expression of response genes through RNA sequencing (RNA-seq) in an attempt to identify the key genes as well as the response pathways under salt stress. These results provide genetic information related to salt stress, which can provide a basis for the further development and evaluation of comprehensive stress-resistant grape rootstock varieties.

## 2. Material and Methods

### 2.1. Plant Material, Stress Application, and Sampling

Grape rootstocks 520A (V. berlandieri × V. riparia), 1103P (V. berlandieri × V. rupestris), 3309C (V. riparia × V. rupestris), 101-14 (V. riparia × V. rupestris), Beta (V. riparia × V. labrusca), and 5BB (V. berlandieri × V. riparia) were used as experimental materials. Among these, 520A, 3309C, and SO4 are salt-tolerant varieties, while the others are intolerant. Two-year-old pot-grown grapevine rootstock plants were obtained from Nanjing Agricultural University (NAU), Nanjing, China, and kept under greenhouse conditions (25 ± 5 °C) at 65% relative humidity (RH) and a 16-h-light and 8-h-dark photoperiod at NAU. The studies were carried out in accordance with relevant institutional, national or international guidelines and regulations. The grapevine plants were kept in a medium of soil, peat, and sand at 3:1:1 (*v*/*v*/*v*) and used as experimental materials. Overall, grape plants with a similar growth status were selected, and a NaCl (130 mmol/L) solution was used for watering for 2 consecutive days to induce salinity stress. The treatment was repeated three times with 6 grapevines in each pot.

During the susceptibility period of the intolerant varieties (P1) and the susceptibility period of the tolerant varieties (P2), we sequenced the transcriptome of these 6 varieties, including the tolerant varieties 3309C (3309C, 3309C-P1, 3309C-P2), 520A (520A, 520A-P1, 520A-P2), and 1103P (1103P, 1103P-P1, 1103P-P2) and the intolerant varieties 5BB (5BB, 5BB-P1), 101-14 (101-14, 101-14-P1), Beta (Beta, Beta-P1). The fourth unfolded leaf from both the treatment and control groups was collected when the plant started to show a stress phenotype. The collected leaf samples were immediately snap-frozen in liquid nitrogen and then stored at −80 °C until further analysis.

### 2.2. RNA Quantification and Qualification

Total RNA of leaf samples was extracted using the CTAB method [34]. RNA degradation and contamination were monitored on 1% agarose gels. RNA purity was checked using the NanoPhotometer spectrophotometer (IMPLEN, Westlake Village, CA, USA). RNA integrity was assessed using the RNA Nano 6000 Assay Kit of the Bioanalyzer 2100 system (Agilent Technologies, Santa Clara, CA, USA). Library preparation and transcriptome sequencing were completed by the Beijing Novogene Technology Corporation (Beijing, China).

### 2.3. Library Preparation for Transcriptome Sequencing

A total amount of 1 μg RNA per sample was used as input material for RNA sample preparation. Sequencing libraries were generated using the NEBNext UltraTM RNA Library Prep Kit for Illumina (New England Biolabs, Inc., Beijing, China) following the manufacturer’s recommendations, and index codes were added to attribute sequences to each sample. Briefly, mRNA was purified from total RNA using poly-T oligo-attached magnetic beads. Fragmentation was carried out using divalent cations under elevated temperature in a NEBNext First Strand Synthesis Reaction Buffer (5X). First-strand cDNA was synthesized using a random hexamer primer and M-MuLV Reverse Transcriptase (RNase H^−^). Second-strand cDNA synthesis was subsequently performed using DNA Polymerase I and RNase H. The remaining overhangs were converted into blunt ends via exonuclease/polymerase activity. After adenylation of the 3′ ends of the DNA fragments, the NEBNext Adaptor with a hairpin loop structure was ligated to prepare the samples for hybridization. In order to select cDNA fragments of preferentially 250~300 bp in length, the library fragments were purified with the AMPure XP system (Beckman Coulter, Beverly, LA, USA). Next, 3 μL of USER Enzyme (NEB, USA) was used with size-selected, adaptor-ligated cDNA at 37 °C for 15 min, followed by 5 min at 95 °C before PCR. The PCR was performed with Phusion High-Fidelity DNA polymerase, universal PCR primers, and the Index (X) Primer. Finally, the PCR products were purified (AMPure XP system) and the library quality was assessed on the Agilent Bioanalyzer 2100 system.

### 2.4. Clustering and Sequencing

Clustering of the index-coded samples was performed on a cBot Cluster Generation System using the TruSeq PE Cluster Kit v3-cBot-HS (Illumia) according to the manufacturer’s instructions. After cluster generation, the library preparations were sequenced on an Illumina Novaseq platform, and 150 bp paired-end reads were generated.

### 2.5. Data Analysis

Raw data (raw reads) in fastq format were firstly processed through in-house Perl scripts. At this step, clean data (clean reads) were obtained by removing reads containing adapters, reads containing poly-N, and low-quality reads from the raw data. All downstream analyses were based on clean high-quality data. HISAT2 software was used to quickly and accurately compare clean reads with the Ensembl reference genome (http://ftp.ensemblgenomes.org/pub/plants/release-47/fasta/vitis_vinifera, accessed on 8 March 2020) to obtain the location information of the reads on the reference genome. Then FPKM of each gene was calculated on the basis of the length of the gene and read counts mapped to this gene. Differentially expressed gene (DEG) analysis was performed using the DESeq R package (1.18.0) [35], and genes with an adjusted *p*-value < 0.05 were considered to be differentially expressed. GO enrichment analysis and the statistical enrichment of DEGs in the KEGG pathways of DEGs were performed with the GOseq R software package and KOBAS software, respectively [36].

### 2.6. Statistical Analysis

Samples were analyzed using statistical analysis of variance (ANOVA) in SPSS 17.0 (SPSS Inc., Chicago, IL, USA), TBtools v1.072 [37], and Origin Pro 9 (Origin Inc., Northampton, MA, USA). All experiments were performed with at least three replicates. For the principal component analysis (PCA) and the heatmap analysis, we used the normalized transcriptome data after taking the logarithm (LogFPKM) and the metabolite content data. MapMan (version 3.6.0RC1, Berlin, Germany) was used to show the differences in the expression of genes involved in various functional modules.

## 3. Results

### 3.1. Salt Treatment Changed the Transcriptome Profile in Grape Rootstocks

We obtained the transcriptomic data of 15 samples under salt treatment with two replicates. An average of 8.435 G of data and 56,478,468 clean reads were obtained for each sample (Table S1). Compared with the grape genome database, the mapping rate of the samples was 88.22%, fully reflecting the changes in the transcription level of different rootstocks under salt treatment conditions. Meanwhile, 1812 new genes were identified in this study (Table S2), which may be used in further functional research.

**Figure 1:**
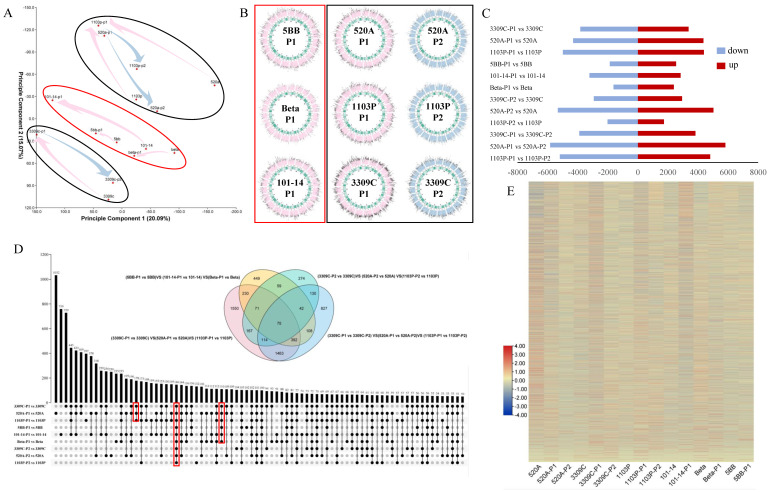
Differentially expressed genes (DEGs) in rootstocks. (**A**) Principal component analysis (PCA): scatterplot of different samples based on the transcriptomic profiles. Pink represents the P1 stage, which is the susceptibility period of intolerant varieties, blue represents the P2 period of the susceptible tolerant varieties, red represents the intolerant varieties, and black represents the tolerant varieties. (**B**) Comparison of the genome expression made by TBtools. In the outer circle, pink represents P1, blue represents P2, and black represents the control group. The heat map in the inner circle represents the distribution of highly expressed genes in the treatment group on the chromosomes. (**C**) Number of up− and downregulated DEGs in samples treated with salt from six rootstocks at two different stages (as shown in Figure 1A). (**D**). DEGs in samples treated with salt from six rootstocks at two different stages (as shown in Figure 1A). The Upset diagram was used to more closely represent the intersection between different temperatures and samples. The green bar graph represents the size of each combination, while a black dot represents yes, a gray dot represents none, and the black bar graph presents the number of intersections. The four−element Venn diagram was used to represent the number of genes with no difference in expression between different groups. Yellow represents DEGs during P1 in intolerant varieties, pink represents DEGs during P1 in tolerant varieties, green represents DEGs during P2 in tolerant varieties, and blue represents differences between P1 and P2 in tolerant varieties. (**E**). Comparison of the transcription level of DEGs using heatmap analysis.

According to the PCA of the transcriptome data (Figure 1A), we observed a strong correlation between tolerant and intolerant varieties. All varieties showed regular changes in the direction of PC1, that is, during P1, the changes were in the negative direction of PC1, and the PC2 changes were in the positive direction. This showed that PC1 is related to the period of salt stress. It can be seen in Figure 1B that the genomes of different rootstock species have basically the same distribution on 19 chromosomes. In the P1 period, the difference in the overall genome expression between the intolerant varieties and the control was much smaller than that of tolerant varieties, while in P2, the difference between tolerant varieties and the control was reduced. In tolerant and intolerant varieties, the distribution of upregulated genes after salt stress was basically the same. In statistics (Figure 1C), 3309C-P1 vs. 3309C, 520A-P1 vs. 520A, 1103P-P1 vs. 1103P, 5BB-P1 vs. 5BB, 101-14-P1 vs. 101-14, Beta-P1 vs. Beta, 3309C-P2 vs. 3309C, 520A-P2 vs. 520A, and 1103P-P2 vs. 1103P enriched 10,000, 11,645, 7720, 3746, 10,344, 5860, 4009, 6061, and 4407 DEGs, respectively. In Figure 1D, 146 genes are present in nine combinations. There are 110 genes present in all combinations of the P1 stage (3309C-P1 vs. 3309C, 520A-P1 vs. 520A, 1103P-P1 vs. 1103P, 5BB-P1 vs. 5BB, 101-14-P1 vs. 101-14, and Beta-P1 vs. Beta) but did not exist in the P2 stage combinations. In total, 178 genes only existed in the P1 of tolerant varieties (3309C-P1 vs. 3309C, 520A-P1 vs. 520A, and 1103P-P1 vs. 1103P). We analyzed the characteristics of the latter two genes (Figure S2) and found that the expression of 178 genes in the P1 of tolerant varieties was significantly higher than that of the control group and the P2 stage. These genes were enriched in biological processes (BP), including monovalent inorganic cation transport, amino acid activation, tRNA aminoacylation, the response to endogenous stimuli, the response to hormones, the response to organic substances, oxoacid metabolic processes, organic acid metabolic processes, glycolipid transport, carbohydrate derivative transport, and lipid transport. In the KEGG pathway analysis, these genes were significantly enriched in autophagy, SNARE interactions in vesicular transport, ascorbate and aldarate metabolism, and inositol phosphate metabolism. The expression levels of 110 genes were downregulated during P1 in tolerant and intolerant varieties, but there was little difference between the P2 stage of tolerant varieties and the control. These genes were extremely significantly enriched in cellular component (CC) processes, including photosystem, photosystem II, photosynthetic membrane, thylakoid, etc., and were extremely significantly enriched in the photosynthesis pathway. This means that the salt treatment firstly affected the photosynthesis of leaves, and tolerant varieties weakened or even eliminated this effect through their own mechanisms in the later stage.

Furthermore, we analyzed the differences between tolerant and intolerant varieties (Figure 1D). In (3309C-P1 vs. 3309C) vs. (520A-P1 vs. 520A) vs. (1103P-P1 vs. 1103P), (5BB-P1 vs. 5BB) vs. (101-14-P1 vs. 101-14) vs. (Beta-P1 vs. Beta), (3309C-P2 vs. 3309C) vs. (520A-P2 vs. 520A) vs. (1103P-P2 vs. 1103P), (3309C- P1 vs. 3309C-P2) vs. (520A-P1 vs. 520A-P2) vs. (1103P-P1 vs. 1103P-P2) 4062, 1426, 932, and 3151 DEGs, respectively, were enriched while 1550, 449, 274, and 827 genes were specifically expressed in a unique combination, and 75 DEGs existed in all four combinations. The genomes of tolerant and intolerant varieties had huge differences in response to salt stress (Figure 1E), which represents the difference in the response to salt stress between these two varieties.

### 3.2. GO Enrichment and KEGG Pathway Analysis of DEGs

**Figure 2:**
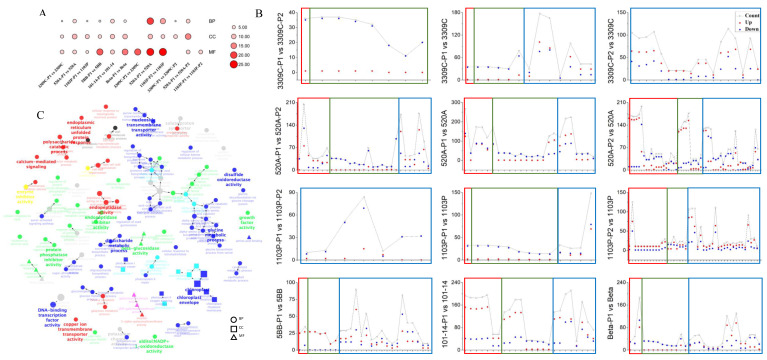
Comparisons of the statistical overrepresentation of GO categories of the three rootstock groups under salt stress. (**A**) Comparison of the total of the three categories of GO enrichment between tolerant and intolerant varieties. (**B**) Comparison of the GO enrichment of tolerant and intolerant varieties. The specific GO terms represented by the abscissa are described in Table S3. The red box represents biological processes (BP), the green box represents cellular components (CC), and the blue box represents molecular functions (MF). (**C**) Visualization of the regulatory network based on the GO analysis results in GO Levels 5–8. The different GO categories are colored according to the correlation between the three groups as shown by the Venn diagram; gray indicates that a term is only enriched but has no specific clusters. Ellipses, squares, and triangles represent biological processes, cell components, and molecular functions, respectively. The size of the shape indicates the number of genes contained in it.

Using ClueGo, we performed GO enrichment analysis on the DEGs of the three groups. Differentially expressed genes were significantly enriched in biological processes (BP), CC, and molecular functions (MF) (Figure 2C). It can be seen from Figure 2A that in the tolerant varieties, the number of GO species enriched in DEGs in P1 was less than that of intolerant varieties, while a large number of MF species were mobilized in P2. From the perspective of the number of genes enriched for each GO term (Figure 2B), the GO enrichment rules of different varieties of the same type are different. Among the salt-tolerant varieties, the DEGs at the P2 stage in 3309C were mainly enriched in MF, and the number of upregulated genes was much higher than that of downregulated genes. Moreover, 520A and 1103P were partially enriched in BPs and CCs, and the number of upregulated genes in several types of BP and CC that were significantly enriched during P2 in 520A was significantly higher than that of downregulated genes. In the P1 stage, the number of downregulated genes in all the enriched CCs was higher than that of upregulated genes. It is worth noting that the DEGs of 1103P in P1 vs. P2 were all enriched in MFs, while 3309C were all enriched in BPs and CCs. This means that the processes of salt-tolerant rootstocks in response to salt stress were not the same. Variety differences also existed in the intolerant varieties. Most of the BPs and CCs that were enriched in DEGs at the P1 stage of 5BB were upregulated, and the number of downregulated genes in 101-14 was higher than that of upregulated genes. The number of downregulated genes in all CCs enriched in Beta was higher than that of upregulated genes. In the P1 stage, the GO pathways shared by the tolerant and intolerant varieties were for photosynthesis, thylakoid-related, DNA binding transcription factor activity, and oxidoreductase activity. P2 added many pathways of MF, such as hydrolase activity, carboxypeptidase activity, and dioxygenase activity.

**Figure 3:**
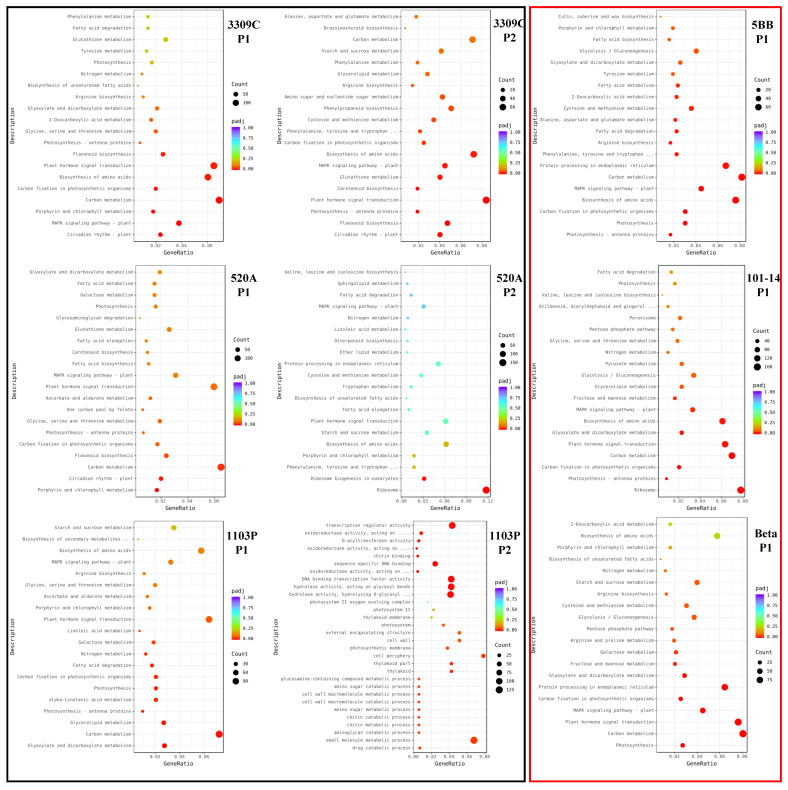
Kyoto Encyclopedia of Genes and Genomes (KEGG) pathways analysis of significantly enriched DEGs in rootstocks. GeneRatio represents the ratio of the number of DEGs annotated to the KEGG pathway to the total number of DEGs. Count represents the number of DEGs annotated to the KEGG pathway. padj represents the *p*-value corrected by multiple hypothesis testing.

Using KEGG pathway analysis, we counted the number of paths for DEGs (Table S4). The tolerant varieties 3309C, 520A, and 1103P were significantly enriched in 10/13, 4/2, and 11/34 KEGG pathways in P1 and P2, respectively, and the intolerant varieties 5BB, 101-14, and Beta were significantly enriched in 19, 10, and 15 KEGG pathways, respectively. Carbon metabolism (vvi01200) was significantly enriched in the P1 stage of tolerant and intolerant varieties, and circadian rhythm—plant (vvi04712) and flavonoid biosynthesis (vvi00941) were only enriched in tolerant varieties. In the intolerant varieties, photosynthesis-related pathways were always the most significantly enriched (Figure 3).

### 3.3. Analysis of Differential Gene Expression Regularity between Tolerant Varieties and Intolerant Varieties

**Figure 4:**
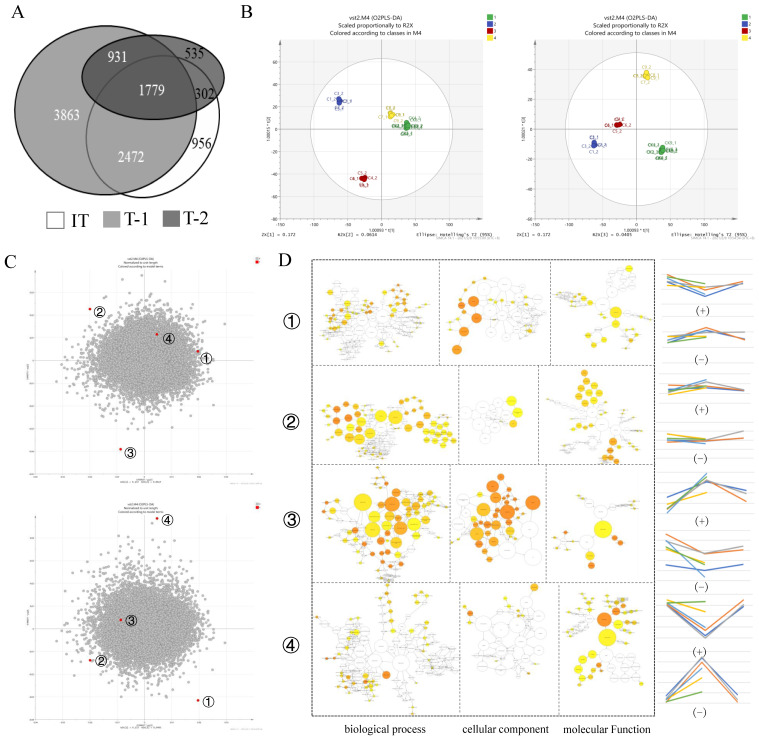
(**A**)**.** Numbers of differentially expressed genes in grape rootstocks grown under salt stress. IT, genes from three intolerant grape rootstock varieties in first stress period; T1, genes from three tolerant grape rootstock varieties in first stress period; T2, genes from three tolerant grape rootstock varieties in second stress period. (**B**). Partial least squares discriminant analysis (PLS−DA) model of the different samples made by SIMCA to analyze the scatter diagram; 1, 2, 3, and 4 represent CK, the two stages of tolerant varieties, and one stage of the intolerant varieties. (**C**): The o2PLS−DA load graph produced by SIMCA. (**D**): DEGs expression patterns and GO enrichment analysis. The right-hand diagram (+) (−) represents the positive correlations and negative correlations, and the line graph represents the expression trend in the samples.

The number and period of DEGs can represent the degree and time of the response of rootstocks with different resistance levels to stress. The different rootstocks all responded quickly to salt stress, and both the resistant and non-resistant rootstocks produced a large amount of DEGs (9045 and 5509, respectively) at the initial stage of stress (Figure 4A). According to partial least squares discriminant analysis (PLS-DA) (Figure 4B,C), the control samples (CK2, CK4, CK3, CK5, CK6, CK9) were gathered together (①), and the P1 samples of the salt-tolerant varieties (C1, C2, C3) were clustered together (②), the P2 stage samples (C7, C8, C9) clustered together (④), and the intolerant varieties at the P1 stage (C4, C5, C6) clustered together (③). In D1–D2, ① and ④ have a close relationship; in D1–D3, ② and ③ have a close relationship. According to the GO function analysis, the DEGs of ① and ③ are mainly used for biological processes and cell components, those of ② are mainly involved in biological processes, and those of ④ are mainly enriched in molecular functions (Figure 4D).

### 3.4. Multiple Stress Pathways Responding to Salt Treatment

**Figure 5:**
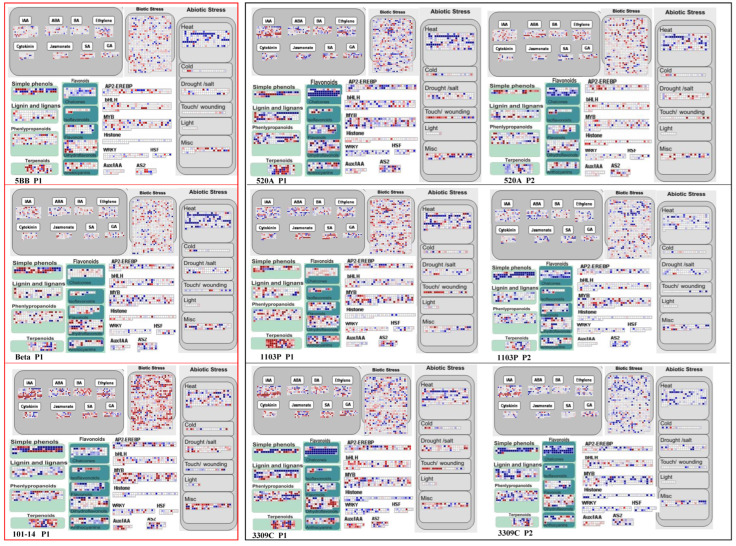
DEGs assigned to the main metabolic process, according to Mapman software. This map consists of the genes that participated in hormone-related genes, abiotic stress, biotic stress, transcription factors, and the secondary metabolism genes including implicated flavonoids, simple phenols, lignin and lignans, phenlypropanoids, and terpenoids. Blue represents upregulation; red represents downregulation.

On the whole, there were great differences in the gene expression of the main signaling pathways related to salt stress in the different varieties (Figure 5). After being exposed to salt stress, most of the biotic stress and some of the abiotic stress genes in 101-14 were downregulated, but there was no such phenomenon in the other two intolerant varieties (5BB and Beta). In particular, the heat stress-related genes in 5BB and Beta showed a significant upregulation trend, whereas heat shock proteins in all varieties showed a significant upregulation trend after exposure to salt stress (Figure S3). Hormones play an important role in the response of rootstocks to salt stress. Most hormone-related genes were downregulated when plants were subjected to salt stress, especially the intolerant varieties (Figure 5). The downward trend of hormones during P2 in tolerant varieties was alleviated to a certain extent. There are many transcription factors in the DEGs, among which AP2-EREBP, bHLH, MYB, Histone, WRKY, HSF, AuxIAA, and AS2 showed the most significant changes (Figure 5). There was little change in WRKY in the intolerant varieties, but this increased during the early stage in tolerant varieties and weakened in the later period. Tolerant varieties mobilized more bHLH and MYB to deal with salt stress than intolerant varieties. However, the changes in transcription factors of the same type in tolerant varieties were also different. The histone of the intolerant variety 520A was downregulated overall under salt stress, while 1103P and 3309C showed an upregulated trend. The changes in secondary metabolism brought about by salt stress were obvious, and most genes related to secondary metabolism were downregulated by salt stress (Figure 5). However, the flavonoid-related pathways had obvious variety characteristics. The tolerant varieties 520A and 3309C showed a significant upward adjustment trend in P1, while 1103P showed a substantial upward adjustment during P2. The intolerant varieties 5BB and 101-14 also showed a slight upward trend. This phenomenon is consistent with the expression levels of *PAL*, *C4H*, and *CHS* in the grape flavonoid pathways (Figure S4).

### 3.5. Response of Genes Related to the Defense System under Salt Stress Treatments

**Figure 6:**
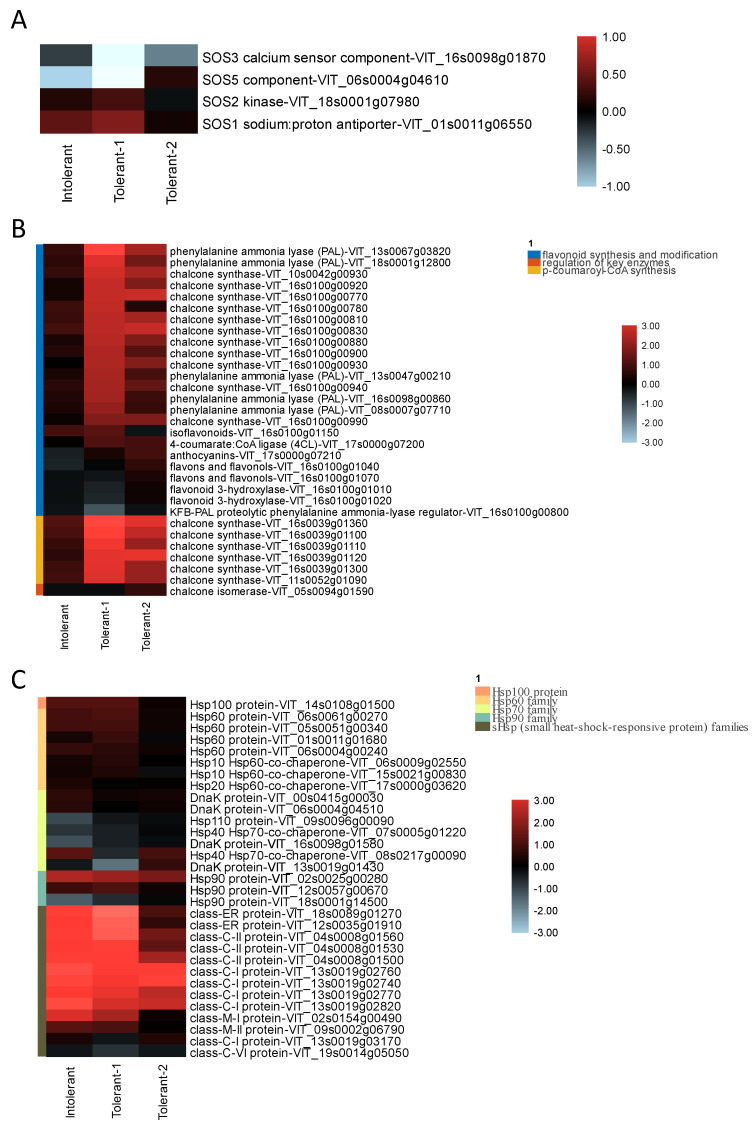
Expression analysis of DEGs related to the defense system under salt stress. Expression analysis of the DEGs involved in (**A**) the SOS gene family, (**B**) phenylpropane metabolism, and (**C**) the HSP gene family under the salt treatment. Each column represents a different tolerance level and each row represents a gene. Red indicates upregulated expression of a DEG and blue indicates downregulated expression. The colors on the left side of the gene are clustered by gene function.

Under salt stress, we first investigated the expression of the SOS (Salt Overly Sensitive) gene. Only in the tolerant rootstock was SOS co-expressed. Here, SOS1 (a sodium–proton antiporter) and SOS2 kinase were upregulated, and the SOS3 (a calcium sensor component) and SOS5 components were downregulated (Figure 6A). The analysis of differences in tolerance showed that the main difference was that the phenylalanine pathway was upregulated in the tolerant varieties (Figure 6B). In total, 31 transcripts were annotated as participating in phenylalanine metabolism, among which *p*-coumaroyl-CoA synthesis contained six transcripts (phenylalanine ammonia lyase (6) and 4-coumarate: CoA ligase (1)) that were upregulated, and 25 transcripts that were related to flavonoid synthesis, of which 22 were upregulated (chalcone synthase (17), dihydroflavonol 4-reductase (1), Type I flavone synthase (2), and isoflavone synthase (1)) and three were downregulated (flavonoid 3-hydroxylase (2) and KFB-PAL proteolytic phenylalanine ammonia-lyase regulator (1)). In the intolerant varieties, only one transcript was upregulated and six transcripts were downregulated. In addition, HSPs also contributed to salinity resistance, and 14 upregulated genes in total were annotated as HSPs (heat shock-responsive proteins) and were upregulated in all rootstocks; nine HSPs (seven upregulated, two downregulated) were in the resistant rootstocks, and eight HSPs (four downregulated and four upregulated) were in the intolerant varieties (Figure 6C).

**Figure 7:**
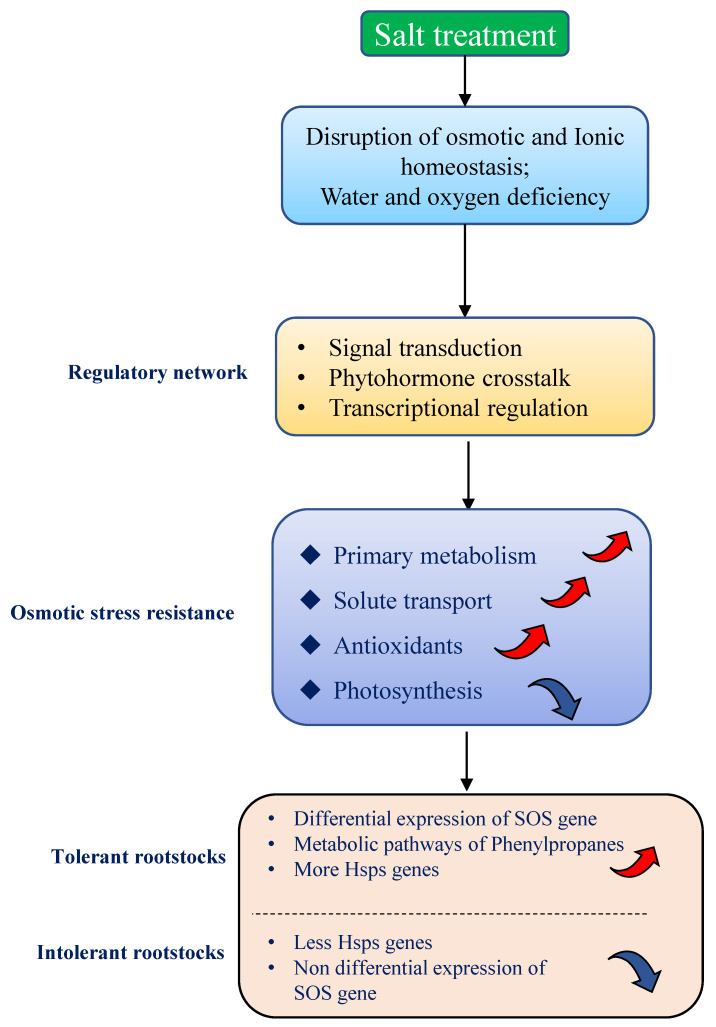
Model of rootstocks in response to salt stress.

We have summarized the physiological, biochemical, and genome-wide transcriptional responses of tolerant and intolerant rootstocks under salt stress (Figure 7). Tolerant rootstocks reduced photosynthesis to increase the antioxidant pathways in order to decrease the production of reactive oxygen species. They also increased their tolerance to abiotic stresses by increasing the synthesis and transport of osmotic substances and improving protein modification and recovery. These findings outline a tolerance mechanism model for rootstocks in coping with osmotic stress, providing important information for improving the resistance of grapes under global climate change.

## 4. Discussion

### 4.1. The Regulation of Plant Salt Tolerance Is a Complex Network

Saline soil will have an osmotic effect on plants, making it more difficult for the roots to extract water. Salt will also create a high salt concentration in the root area to produce ion toxicity, which affects plant development, metabolic adaptation, and ion chelation or rejection [10]. Plant roots absorb water from the soil, absorb Na^+^ and other ions, and transport these ions to the leaves through transpiration. As the water evaporates, large amounts of salt accumulate in the apoplasts and other cell compartments [38]. Na^+^ mainly affects the photosynthesis of plants. Studies have shown that excessive Na^+^ can affect photosynthesis by destroying the proton motive force and chloroplast function, as well as interfering with CO_2_ immobilization enzymes [39]; in addition, excessive Na^+^ will also affect plants. The lack of absorption of other cations, especially K^+^, and the molecular similarity of potassium makes it more easily replaced by sodium. However, Na^+^ cannot perform the biological function of K^+^, and thus the cell balance between sodium and potassium is particularly important for the survival of plants in saline soil [40,41,42].

Plants’ salt tolerance is a complex process involving multiple genes and multiple pathways of induction. A large number of studies have shown that salt tolerance in plants is mainly through the following regulatory pathways: the reactive oxygen species (ROS) signal-mediated mitogen-activated protein kinase (MAPK) pathway; the NAC (NAM, ATAF, CUC) pathway; the Ca^2+^ signal-mediated salt hypersensitivity (SOS) pathway; the endoplasmic reticulum-related protein degradation (ERAD) pathway; and the abscisic acid (ABA), brassinolide (BR), and other plant hormone-mediated salt stress response, etc. [43,44]. Salt stress affects physiological and biochemical changes [28], which was also found in this study in the transcriptome analysis showing that the changes in secondary metabolism brought about by salt stress are obvious, and most of the related genes were downregulated. Under salt stress conditions, the enzyme activity in plants decreases, active oxygen accumulates, cell division and extension are inhibited, cell membranes are damaged, osmotic balance is disrupted, and ultimately growth is inhibited [45]. These activities were also confirmed in this study. Hormones play an important role in the response of rootstocks to salt stress. In our study, most hormone-related genes were down-regulated when plants were subjected to salt stress, and the downward trend during P2 in tolerant varieties was alleviated to a certain extent. Among the hormones, jasmonates (JAs) are positive regulators of salt tolerance [46]. Ahmed et al. [47] proved that jasmonate signalling is a central element of both biotic and abiotic stress responses, and exogenous jasmonate can rescue growth in salt-sensitive cell lines.

Stress-responsive TFs function in conjunction with the promoter regions to regulate the expression of the salt stress-responsive genes involved in salt tolerance [9]. In our study, TFs AP2-EREBP, bHLH, MYB, Histone, WRKY, HSF, AuxIAA, and AS2 had the most significant changes. For example, there was little change in WRKY in intolerant varieties, but it was increased during the early stage in tolerant varieties and weakened in the later period. Overexpression of the MYB transcription factor (MYB48-1) in rice also enhanced the drought and salt stress responses induced by mannitol and propylene glycol [14]. In grapes, we found that tolerant varieties mobilized more bHLH and MYB to deal with salt stress than intolerant varieties, but MYB plays a key role that needs further study. It is worth mentioning that in this study, the genes of heat shock proteins were upregulated in the tolerant varieties. Salt stress is associated with the rapid production of reactive oxygen species (ROS) in plants, while HSFs might function as ROS-dependent redox sensors [48]. In *Arabidopsis*, MPK3 and MPK6 phosphorylate and activate HSFA4A, thereby controlling ROS homeostasis and positively regulating salt-stress responses [49]. This indicates that HSFs may play an important role in the response of grapes to salt stress.

Plants mainly respond to salt stress by adjusting the ion balance in the cell to maintain a high K^+^/Na^+^ ratio in the cytoplasm [50]. If we take the SOS pathway as an example, salt stress induces the accumulation of Ca^2+^ in the cytoplasm. The Ca^2+^ receptor SOS3 protein of the calcineurin-like B (CBL) family and the CBL protein kinase (CIPK) family SOS2 form the SOS3–SOS2 complex, which positively regulates the Na^+^/H^+^ antiporter (NHX) to transport Na^+^ from the cytoplasm to the vacuole, and phosphorylates the H^+^/cation antiporter to transport sodium out of the cell, helping plants maintain intracellular ion homeostasis and reducing stress damage [11,12,49]. In this study, SOS1 and SOS2 were upregulated, and the SOS3 and SOS5 components were downregulated.

### 4.2. Research on Grape’s Salt Tolerance System Is Attracting More and More Attention

Grapes need the right salt concentration to grow, and if they exceed a certain threshold, they will suffer stress and injury [10]. Grapes are facing the threat of salt stress in the environment, causing osmotic stress, ion toxicity, pH damage, and reactive oxygen stress, affecting their external morphology, photosynthesis, ion balance, and membrane permeability [10,51]. At present, research on the molecular mechanisms of plants’ salt tolerance has mainly focused on model crops such as *Arabidopsis thaliana*, while there are relatively few studies on perennial woody fruit tree crops. The response of grapes to salt stress is divided into two stages: the first stage is characterized by osmotic stress due to reduced grape growth, increased free radical and active oxygen content, and the synthesis of osmotic regulator substances, superoxide dismutase (SOD), and peroxide. Protective enzymes such as enzymes (POD) and catalase (CAT) stimulate the corresponding protective mechanisms [52,53]. The second stage is the ion toxicity stage, when the photosynthetic gas exchange decreases, causing the grapes to fail to grow normally. Plants restrict Na^+^ absorption through selective absorption, separating Na^+^ into vacuoles and activating Na^+^ to the apoplast space to minimize the accumulation of Na^+^ in the cytoplasm [54,55]. Among these mechanisms, separating Na^+^ into vacuoles can effectively reduce the osmotic potential and avoid water shortages in plants. This process is mediated by NHX protein [56,57,58].

Research on the salt tolerance of grape rootstocks started relatively late, and there are few studies on its salt tolerance mechanism [26,59,60]. In grape, excessive Cl^-^ is also toxic. Research by Walker [29] has shown that the effects of salt stress are more related to the concentration of chloride ions. Rootstocks can increase salt tolerance by restricting Na or Cl from entering the buds or by chelating sodium in the vacuoles of old leaves [61,62]. These phenomena correspond to the increased activity of anion transmembrane transport and potassium ion transmembrane transport observed in the resistant varieties in this study. Tolerant and intolerant rootstocks have different mechanisms to respond to salt stress. This study confirmed that tolerant varieties mobilized more genes than intolerant varieties to resist salt stress in the early stage of salt stress. Rootstocks alleviate the transcriptional response of genes involved in carbohydrate and amino acid metabolism. Tolerant rootstocks further reduce photosynthesis and increase the antioxidant pathways, thereby reducing the production of reactive oxygen species. They also increase tolerance to abiotic stresses by increasing the synthesis and transport of osmotic substances and by improving protein modification and recovery. The results provide a practical evaluation of the resistance of grape rootstocks, giving significant evidence on the adaptability of different grape rootstocks to stress, as well as a theoretical basis for resistant rootstock breeding.

## 5. Conclusions

Grapes are moderately sensitive to salinity, and the utilization of rootstocks is currently the most effective way for grapes to deal with abiotic stress. Salt stress has a negative effect on grape growth. Firstly, it affects the photosynthesis of leaves, and tolerant varieties weaken or even eliminate this effect through their own mechanisms in the later stage. Tolerant rootstocks reduce photosynthesis to increase the antioxidant pathways in order to decrease the production of reactive oxygen species. They also increase their tolerance to abiotic stresses by increasing the synthesis and transport of osmotic substances and by improving protein modification and recovery. Salt stress affects the expression of genes related to plant abiotic stress, biotic stress, transcription factors, hormones, and secondary metabolism. Tolerant varieties mobilized more bHLH, WRKY, and MYB transcription factors to respond to salt stress than intolerant varieties. Moreover, SOSs were co-expressed in the tolerant rootstocks.

## Data Availability

All raw data reported in this article have been deposited in the National Center for Biotechnology Information sequence read archive (transcriptome sequencing accession SRA accession number: PRJNA764545). All data supporting this research result can be obtained in the article and within its Supplementary Materials published online. Additional data related to this article may be requested from the authors.

## Supplementary Materials

The following supporting information can be downloaded at: https://www.mdpi.com/article/10.3390/plants11091167/s1, Figure S1: The effect of salt treatment on the endogenous substances of the rootstocks of tolerant varieties and intolerant varieties; Figure S2: The expression and function analysis of two types of differential genes; Figure S3: DEGs assigned to abiotic stress based on Mapman software; Figure S4: Effect of salt treatment on the genes involved in general phenylpropanoid pathway in grapes; Table S1: Mapping statistics of RNA-Seq; Table S2: New genes identified in this study; Table S3: GO analysis; Table S4: KEGG pathways.

## References

[1] Bailey-Serres J., Fukao T., Gibbs D.J., Holdsworth M.J., Lee S.C., Licausi F., Perata P., Voesenek L.A., van Dongen J.T. (2012). Making sense of low oxygen sensing. Trends Plant Sci..

[2] Keller M. (2010). Managing grapevines to optimise fruit development in a challenging environment: A climate change primer for viticulturists. Aust. J. Grape Wine Res..

[3] Miras-Avalos J.M., Intrigliolo D.S. (2017). Grape Composition under Abiotic Constrains: Water Stress and Salinity. Front. Plant Sci..

[4] Hasegawa P.M., Bressan R.A., Zhu J.K., Bohnert H.J. (2000). Plant Cellular and Molecular Responses to High Salinity. Annu. Rev. Plant Physiol. Plant Mol. Biol..

[5] Zhu J.K. (2002). Salt and drought stress signal transduction in plants. Annu. Rev. Plant Biol..

[6] Lowe K.M., Walker M.A. (2006). Genetic linkage map of the interspecific grape rootstock cross Ramsey (*Vitis champinii*) × Riparia Gloire (*Vitis riparia*). Theor. Appl. Genet..

[7] Riaz S., Pap D., Uretsky J., Laucou V., Boursiquot J.M., Kocsis L., Andrew Walker M. (2019). Genetic diversity and parentage analysis of grape rootstocks. Theor. Appl. Genet..

[8] Ferris H., Zheng L., Walker M.A. (2012). Resistance of Grape Rootstocks to Plant-parasitic Nematodes. J. Nematol..

[9] Rivero R.M., Ruiz J.M., Romero L. (2003). Role of grafting in horticultural plants under stress conditions. J. Food Agric. Environ..

[10] Serra I., Strever A., Myburgh P., Deloire A. (2014). The interaction between rootstocks and cultivars (*Vitis vinifera* L.) to enhance drought tolerance in grapevine. Aust. J. Grape Wine Res..

[11] Jogaiah S., Ramteke S.D., Sharma J., Upadhyay A.K. (2014). Moisture and salinity stress induced changes in biochemical constituents and water relations of different grape rootstock cultivars. Int. J. Agron..

[12] Wani S.H., Kumar V., Shriram V., Sah S.K. (2016). Phytohormones and their metabolic engineering for abiotic stress tolerance in crop plants. Crop J..

[13] Parida A.K., Das A.B. (2005). Salt tolerance and salinity effects on plants: A review. Ecotoxicol. Environ. Saf..

[14] Wani S.H., Kumar V., Khare T., Guddimalli R., Parveda M., Solymosi K., Suprasanna P., Kavi Kishor P.B. (2020). Engineering salinity tolerance in plants: Progress and prospects. Planta.

[15] Munns R., Tester M. (2008). Mechanisms of salinity tolerance. Annu. Rev. Plant Biol..

[16] Halfter U., Ishitani M., Zhu J.-K. (2000). The Arabidopsis SOS_2_ protein kinase physically interacts with and is activated by the calcium-binding protein SOS_3_. Proc. Natl. Acad. Sci. USA.

[17] Ji H., Pardo J.M., Batelli G., Van Oosten M.J., Bressan R.A., Li X. (2013). The Salt Overly Sensitive (SOS) pathway: Established and emerging roles. Mol. Plant.

[18] Zhang T., Chen S., Harmon A.C. (2014). Protein phosphorylation in stomatal movement. Plant Signal. Behav..

[19] Xiong H., Li J., Liu P., Duan J., Zhao Y., Guo X., Li Y., Zhang H., Ali J., Li Z. (2014). Overexpression of OsMYB48-1, a novel MYB-related transcription factor, enhances drought and salinity tolerance in rice. PLoS ONE.

[20] Daldoul S., Guillaumie S., Reustle G.M., Krczal G., Ghorbel A., Delrot S., Mliki A., Hofer M.U. (2010). Isolation and expression analysis of salt induced genes from contrasting grapevine (*Vitis vinifera* L.) cultivars. Plant Sci..

[21] Jellouli N., Ben Jouira H., Skouri H., Ghorbel A., Gourgouri A., Mliki A. (2008). Proteomic analysis of Tunisian grapevine cultivar Razegui under salt stress. J. Plant Physiol..

[22] Tillett R.L., Ergul A., Albion R.L., Schlauch K.A., Cramer G.R., Cushman J.C. (2011). Identification of tissue-specific, abiotic stress-responsive gene expression patterns in wine grape (*Vitis vinifera* L.) based on curation and mining of large-scale EST data sets. BMC Plant Biol..

[23] Wen Y., Wang X., Xiao S., Wang Y. (2012). Ectopic expression of VpALDH2B4, a novel aldehyde dehydrogenase gene from Chinese wild grapevine (*Vitis pseudoreticulata*), enhances resistance to mildew pathogens and salt stress in Arabidopsis. Planta.

[24] Tak H., Mhatre M. (2013). Cloning and molecular characterization of a putative bZIP transcription factor VvbZIP23 from *Vitis vinifera*. Protoplasma.

[25] Tak H., Mhatre M. (2013). Molecular characterization of VvSDIR1 from *Vitis vinifera* and its functional analysis by heterologous expression in Nicotiana tabacum. Protoplasma.

[26] Alizadeh M., Singh S., Patel V., Bhattacharya R., Yadav B.P. (2010). In vitro responses of grape rootstocks to NaCl. Biol. Plant..

[27] Suarez D.L., Celis N., Anderson R.G., Sandhu D. (2019). Grape Rootstock Response to Salinity, Water and Combined Salinity and Water Stresses. Agronomy.

[28] Walker R., Blackmore D.H., Clingeleffer P.R., Tarr C.R. (2007). Rootstock effects on salt tolerance of irrigated field-grown grapevines (*Vitis vinifera* L. cv. Sultana): 3. Fresh fruit composition and dried grape quality. Aust. J. Grape Wine Res..

[29] Walker R.R., Blackmore D.H., Clingeleffer P.R., Correll R.L. (2004). Rootstock effects on salt tolerance of irrigated field-grown grapevines (*Vitis vinifera* L. cv. Sultana): 2. Ion concentrations in leaves and juice. Aust. J. Grape Wine Res..

[30] Walker R.R., Blackmore D.H., Clingeleffer P.R., Correll R.L. (2002). Rootstock effects on salt tolerance of irrigated field-grown grapevines (*Vitis vinifera* L. cv. Sultana): 1. Yield and vigour inter-relationships. Aust. J. Grape Wine Res..

[31] Upadhyay A., Upadhyay A.K., Bhirangi R.A. (2012). Expression of Na^+^/H^+^ antiporter gene in response to water and salinity stress in grapevine rootstocks. Biol. Plant..

[32] Mehanna H.T., Fayed T.A., Rashedy A.A. (2010). Response of two grape rootstocks to some salt tolerance treatments under saline water conditions. J. Hortic. Sci. Ornam. Plants.

[33] Wooldridge J., Olivier M. (2014). Effects of weathered soil parent materials on Merlot grapevines grafted onto 110 Richter and 101-14Mgt rootstocks. S. Afr. J. Enol. Vitic..

[34] Wang C., Han J., Shangguan L., Yang G., Kayesh E., Zhang Y., Leng X., Fang J. (2014). Depiction of grapevine phenology by gene expression information and a test of its workability in guiding fertilization. Plant Mol. Biol. Rep..

[35] Benjamini Y., Hochberg Y. (1995). Controlling the false discovery rate: A practical and powerful approach to multiple testing. J. R. Stat. Soc. Ser. B.

[36] Zhang Z., Zhao P., Zhang P., Su L., Jia H., Wei X., Fang J., Jia H. (2020). Integrative transcriptomics and metabolomics data exploring the effect of chitosan on postharvest grape resistance to Botrytis cinerea. Postharvest Biol. Technol..

[37] Chen C., Chen H., Zhang Y., Thomas H.R., Frank M.H., He Y., Xia R. (2020). TBtools: An Integrative Toolkit Developed for Interactive Analyses of Big Biological Data. Mol. Plant.

[38] Brini F., Masmoudi K. (2012). Ion Transporters and Abiotic Stress Tolerance in Plants. ISRN Mol. Biol..

[39] Bose J., Munns R., Shabala S., Gilliham M., Pogson B., Tyerman S.D. (2017). Chloroplast function and ion regulation in plants growing on saline soils: Lessons from halophytes. J. Exp. Bot..

[40] Benito B., Haro R., Amtmann A., Cuin T.A., Dreyer I. (2014). The twins K^+^ and Na^+^ in plants. J. Plant Physiol..

[41] Haider M.S., Jogaiah S., Pervaiz T., Yanxue Z., Khan N., Fang J. (2019). Physiological and transcriptional variations inducing complex adaptive mechanisms in grapevine by salt stress. Environ. Exp. Bot..

[42] Sharma D.K., Dubey A., Srivastav M., Singh A., Sairam R., Pandey R., Dahuja A., Kaur C. (2011). Effect of putrescine and paclobutrazol on growth, physiochemical parameters, and nutrient acquisition of salt-sensitive citrus rootstock Karna khatta (Citrus karna Raf.) under NaCl stress. J. Plant Growth Regul..

[43] Cui F., Liu L., Zhao Q., Zhang Z., Li Q., Lin B., Wu Y., Tang S., Xie Q. (2012). Arabidopsis ubiquitin conjugase UBC32 is an ERAD component that functions in brassinosteroid-mediated salt stress tolerance. Plant Cell.

[44] Tardieu F., Simonneau T., Muller B. (2018). The Physiological Basis of Drought Tolerance in Crop Plants: A Scenario-Dependent Probabilistic Approach. Annu. Rev. Plant Biol..

[45] Tuteja N. (2007). Mechanisms of high salinity tolerance in plants. Methods Enzym..

[46] Kazan K. (2015). Diverse roles of jasmonates and ethylene in abiotic stress tolerance. Trends Plant Sci..

[47] Ismail A., Riemann M., Nick P. (2012). The jasmonate pathway mediates salt tolerance in grapevines. J. Exp. Bot..

[48] Miller G.A.D., Mittler R.O.N. (2006). Could Heat Shock Transcription Factors Function as Hydrogen Peroxide Sensors in Plants?. Ann. Bot..

[49] Yang Y., Guo Y. (2018). Unraveling salt stress signaling in plants. J. Integr. Plant Biol..

[50] Munns R. (2002). Comparative physiology of salt and water stress. Plant Cell Environ..

[51] Yin R., Bai T., Ma F., Wang X., Li Y., Yue Z. (2010). Physiological responses and relative tolerance by Chinese apple rootstocks to NaCl stress. Sci. Hortic..

[52] He L., Ban Y., Inoue H., Matsuda N., Liu J., Moriguchi T. (2008). Enhancement of spermidine content and antioxidant capacity in transgenic pear shoots overexpressing apple spermidine synthase in response to salinity and hyperosmosis. Phytochemistry.

[53] Tavakkoli E., Fatehi F., Coventry S., Rengasamy P., McDonald G.K. (2011). Additive effects of Na^+^ and Cl^−^ ions on barley growth under salinity stress. J. Exp. Bot..

[54] Yamaguchi T., Hamamoto S., Uozumi N. (2013). Sodium transport system in plant cells. Front. Plant Sci..

[55] Maathuis F.J., Ahmad I., Patishtan J. (2014). Regulation of Na^+^ fluxes in plants. Front. Plant Sci..

[56] Kronzucker H.J., Britto D.T. (2011). Sodium transport in plants: A critical review. New Phytol..

[57] Bassil E., Ohto M.A., Esumi T., Tajima H., Zhu Z., Cagnac O., Belmonte M., Peleg Z., Yamaguchi T., Blumwald E. (2011). The Arabidopsis intracellular Na^+^/H^+^ antiporters NHX5 and NHX6 are endosome associated and necessary for plant growth and development. Plant Cell.

[58] Aylagas E., Borja A., Pochon X., Zaiko A., Keeley N., Bruce K., Hong P., Ruiz G.M., Stein E.D., Theroux S. (2020). Translational Molecular Ecology in practice: Linking DNA-based methods to actionable marine environmental management. Sci. Total Environ..

[59] Walker R.R., Read P.E., Blackmore D.H. (2000). Rootstock and salinity effects on rates of berry maturation, ion accumulation and colour development in Shiraz grapes. Aust. J. Grape Wine Res..

[60] Sohrabi S., Ebadi A., Jalali S., Salami S.A. (2017). Enhanced values of various physiological traits and VvNAC1 gene expression showing better salinity stress tolerance in some grapevine cultivars as well as rootstocks. Sci. Hortic..

[61] Paranychianakis N., Angelakis A. (2008). The effect of water stress and rootstock on the development of leaf injuries in grapevines irrigated with saline effluent. Agric. Water Manag..

[62] Tregeagle J.M., Tisdall J., Blackmore D., Walker R. (2006). A diminished capacity for chloride exclusion by grapevine rootstocks following long-term saline irrigation in an inland versus a coastal region of Australia. Aust. J. Grape Wine Res..

