# Supplementary material for: Transcriptomic Analysis Elaborates the Resistance Mechanism of Grapevine Rootstocks against Salt Stress
Source: Plants (Basel). 2022 Apr 26;11(9):1167. doi: 10.3390/plants11091167 (PMC9103662; doi:10.3390/plants11091167)
Supplement: Supplementary file 1 [file plants-11-01167-s001.zip › Supplement Figures.pdf]

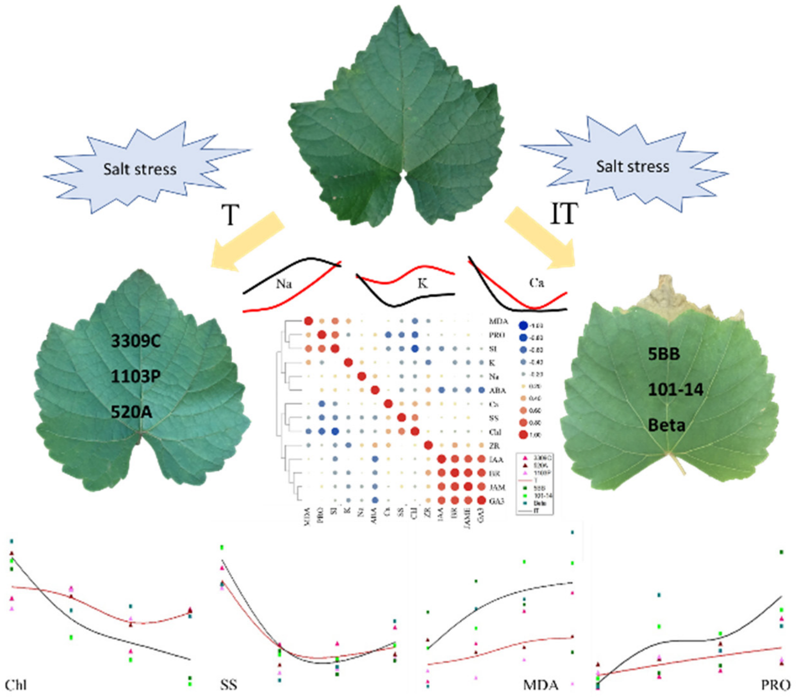

Figure S1 The effect of salt treatment on the endogenous substances of the rootstocks of tolerant varieties and intolerant varieties. The red line represents the tolerant varieties (T), and the black line represents the intolerant varieties.

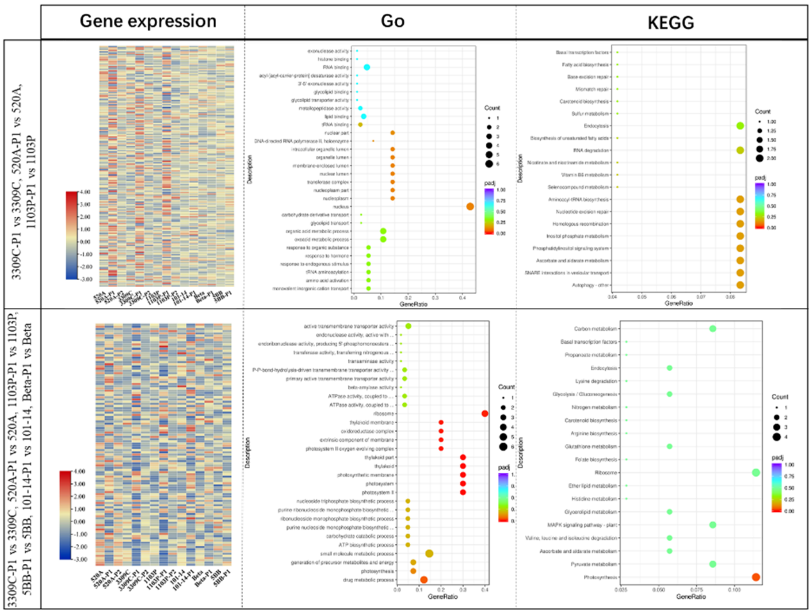

Figure S2. The expression and function analysis of two types of differential genes, including 178 genes (3309C-P1 vs 3309C, 520A-P1 vs 520A, 1103P-P1 vs 1103P) that exist only at P1 stage of tolerant varieties; 110 genes only exist all combinations of P1 stage (3309C-P1 vs 3309C, 520A-P1 vs 520A, 1103P-P1 vs 1103P, 5BB-P1 vs 5BB, 101-14-P1 vs 101-14, Beta-P1 vs Beta) but not in P2.

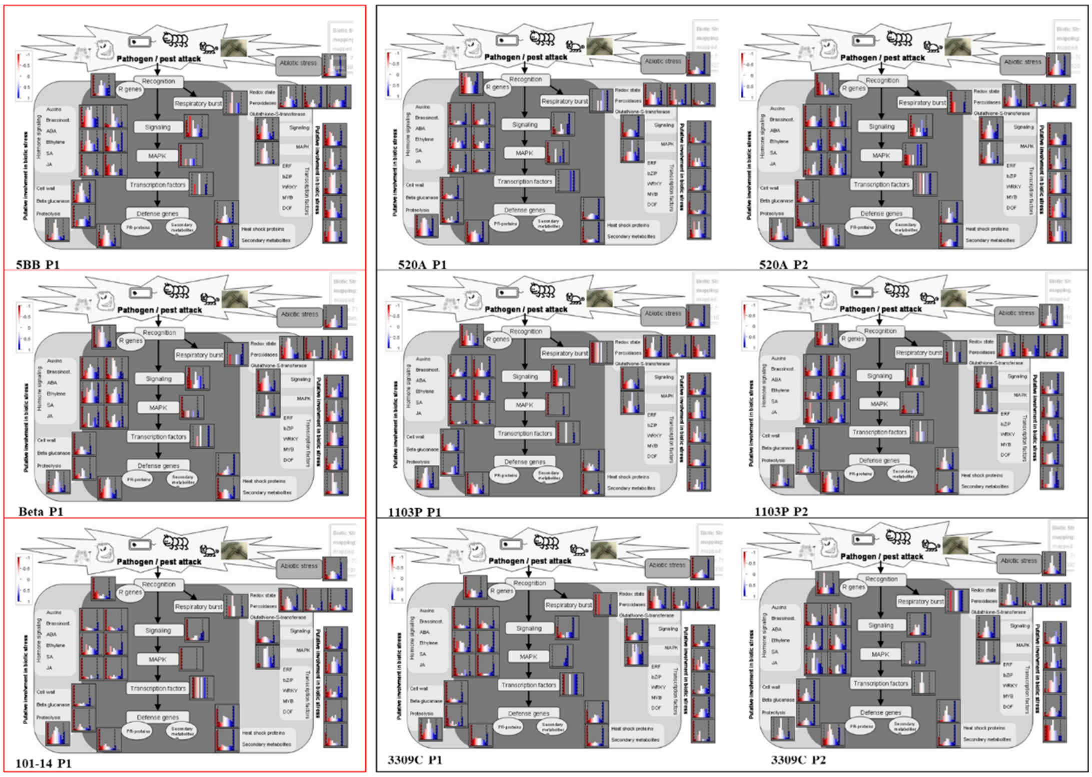

Figure S3. DEGs assigned to abiotic stress based on Mapman software. This map consisted of the genes that participated in abiotic stress, redox, signaling, transcription factors, heat shock proteins, pathogenesis protein, hormone relative genes, as well as the genes implicated in cell wall and proteolysis. Blue represents upregulation, while red represents downregulation.

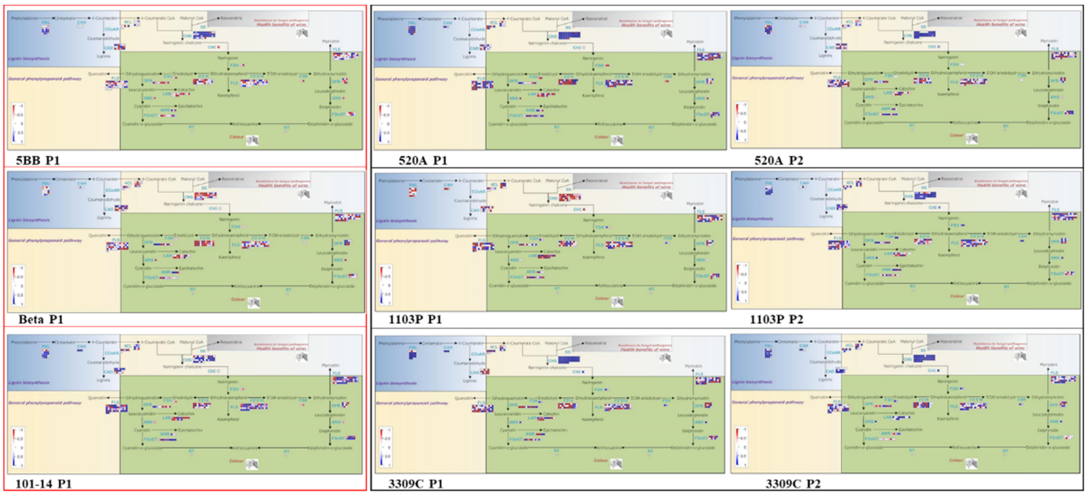

Figure S4. Effect of salt treatment on the genes involved in general phenylpropanoid pathway in grapes. The relative transcription levels of different genes in treated samples with salt treatment compared to control were displayed in the heatmap. Blue represents downregulation, while red represents upregulation. PAL, C4H, 4CL, CCoAR, CAD, CHS, FLS, DFR, ANS, F3oGT, F3H, F3’H, and F3’5’H represent phenylalanine ammonialyase, cinnamate-4-hydroxylase, 4-coumaroyl-coA synthase, cinnamoyl-CoA reductase, cinnamyl alcohol dehydrogenase, chalcone synthase, flavonol synthase, dihydroflavonol reductase, anthocyanidin synthase, flavonol-3-O-glucosyl transferase, flavanone 3-hydroxylase, flavonoid 3'-hydroxylase, and flaconoid-3',5'-hydroxylase, respectively.
